# Dietary calcium intake in primary hyperparathyroidism and in its normocalcemic variant: a case-control study

**DOI:** 10.3389/fendo.2024.1428640

**Published:** 2025-02-03

**Authors:** Nicolò Bisceglia, Matteo Malagrinò, Anna Piazza, Giulia Vandi, Andrea Repaci, Uberto Pagotto, Guido Zavatta

**Affiliations:** ^1^ Division of Endocrinology and Diabetes Prevention and Care, IRCCS Azienda Ospedaliero-Universitaria di Bologna, Bologna, Italy; ^2^ Department of Medical and Surgical Sciences (DIMEC), Alma Mater Studiorum University of Bologna, Bologna, Italy

**Keywords:** calcium intake, normocalcemic primary hyperparathyroidism, nutrition, parathyroid tumor, parathyroidectomy, osteoporosis, surgery, guidelines

## Abstract

**Introduction:**

Normocalcemic primary hyperparathyroidism (NHPT) is considered to be an early stage in the evolution of primary hyperparathyroidism (PHPT). To formulate a correct diagnosis, secondary hyperparathyroidism due to low calcium intake must be excluded. Whether dietary calcium intake might affect the clinical presentation of PHPT or NHPT has never been addressed consistently.

**Objective:**

To describe patients with a diagnosis of NHPT or PHPT in relation to their calcium intake, through three standard validated questionnaires; to describe clinical, biochemical and radiological features of NHPT and PHPT patients compared to each other and to a control group.

**Design:**

Cross-sectional study.

**Setting:**

Outpatient, single academic medical center.

**Patients:**

109 consecutive women recruited from February 2021 through April 2023. 54 patients with mild primary hyperparathyroidism (PHPT or NHPT) were age-matched with 55 unselected women undergoing bone density test screening due to recently diagnosed hormone-positive breast cancer. NHPT diagnosis was based on multiple determinations of both total and albumin-corrected serum calcium.

**Interventions:**

Administration of all the following during routine endocrine consultation: a country-specific food-frequency questionnaire (LOC), the International Osteoporosis Foundation Calcium Calculator (IOF) and the National Osteoporosis Foundation calcium questionnaire (NOF).

**Main outcome measures:**

Any association between dietary calcium intake and clinical, radiological, or biochemical features.

**Results:**

All three questionnaires confirmed that NHPT patients had similar calcium intake as those with PHPT or controls. Biochemistries and bone turnover markers were similar between the two variants of hyperparathyroidism, except for serum calcium (sCa). NHPT patients had a significantly lower BMD and T-score at one-third distal radius compared to PHPT, while the prevalence of nephrolithiasis and clinical fractures were similar. Multivariate analysis investigating predictors of serum calcium showed that age, eGFR, calcium intake and 25(OH)D did not significantly affect serum calcium, while multivariate analysis investigating predictors of PTH (age, variant NHPT vs. PHPT, eGFR, calcium intake, 25(OH)D, cholecalciferol supplements) showed that calcium intake, variant and renal function, significantly influenced PTH levels.

**Conclusions:**

All patients with primary hyperparathyroidism, particularly those with low dietary calcium intake, should be advised not to restrict dietary calcium to prevent further increase in PTH levels. Whether maintaining adequate calcium intake might positively impact bone density or biochemistries in patients refraining from surgery, should be addressed in longitudinal studies.

## 1 Introduction

Classical primary hyperparathyroidism (PHPT) is the third most common endocrine disorder, as well as the most frequent cause of hypercalcemia in ambulatory patients (1). This disease is diagnosed in the setting of hypercalcemia with elevated or inappropriately normal PTH levels. Another variant or subtype of primary hyperparathyroidism is called normocalcemic primary hyperparathyroidism (NHPT), which is characterized by serum calcium persistently in the normal range, and PTH persistently elevated, after excluding any obvious or underlying causes (2). The whole notion about NHPT as an “entity” arose because of routinely PTH testing for conditions other than PHPT, in which instead hypercalcemia is the required characteristic before PTH is tested. PTH is ordered routinely in the investigation of various bone and mineral disorders, and in most, it is normal (3–5). NHPT, when correctly diagnosed, appears as a much rarer form of primary hyperparathyroidism, and can also be considered as an early stage in the natural history of classical primary hyperparathyroidism (PHPT) (6, 7).

In order to formulate a correct diagnosis of NHPT, secondary hyperparathyroidism due to insufficient calcium intake must be excluded (8, 9). Other than that, whether calcium intake might affect the clinical or biochemical presentation of PHPT or NHPT has never been addressed consistently in the literature. In this regard two recent major studies (10, 11) compare the two variant in detail but do not investigate the dietary calcium intake. Over twenty years ago, a few studies were designed to investigate the relationship between calcium intake and hyperparathyroidism, with conflicting results (12–16). Net of these considerations many clinicians advise their patients with classical PHPT (i.e., with hypercalcemia) to restrict their calcium intake, most likely for fear of aggravating hypercalcemia or hypercalciuria. The latest guidelines on the management of primary hyperparathyroidism (2) state that due to lack of evidence, it seems prudent to recommend patients with PHPT receive the same amount of dietary calcium as that suggested for the general population, as reported by the Institute of Medicine nutritional guidelines for calcium (17) and also recommend further studies about potential role of diet on clinical manifestations of PHPT.

A very large prospective cohort study (Nurses’ Health Study I) examined the association between calcium intake and subsequent risk of PHPT in women. More than 58,000 women without a history of underlying PHPT were followed for over a 22-year period and divided into 5 groups according to dietary calcium intake. The relative risk of PHPT was reduced (RR 0.56, 0.37-0.86, p = 0.009) in the group with the highest quintile of dietary calcium intake compared to the lowest quintile, therefore, these authors postulated that women with increased dietary or supplemental calcium intake had a reduced risk of developing PHPT independent of age, waist circumference, diet, and other factors (18). A period of several years of suboptimal calcium intake might lead to persistent PTH release. This mechanism would represent a trigger for the proliferation of the parathyroid cell and could therefore increase the probability of newborn somatic mutations and eventual parathyroid autonomy (19). This observational study, however, did not prove causality. In addition, several variables were not independently accounted for in the evaluation of the outcome. The highest quintile of calcium intake, for example, was also associated with the highest intake of vitamin D and proteins.

The major determinants of serum calcium are the change in “set-point” of the abnormal parathyroid tissue, age, renal function, and 25(OH)D levels. Presumably, a component of the serum calcium level might also be dependent on intestinal calcium absorption, because 1,25-dihydroxyvitamin D [1,25(OH)2D] levels are stimulated by PTH. In healthy individuals, it is known that low dietary calcium intake tends to cause lower serum and urinary calcium levels, with consequent stimulation of PTH secretion and 1,25(OH)2D synthesis, with eventual increase in intestinal calcium absorption. By contrast, high dietary calcium intake has been shown to have a suppressive effect on PTH (20, 21).

In PHPT, the abnormal parathyroid tissue, is generally thought to maintain a certain responsiveness to serum calcium fluctuations and could respond to calcium restriction with a further increase in PTH synthesis and a potential worsening of the clinical manifestations of the disease, thereby exacerbating bone loss, in turn leading to an even higher calcium load to be filtered through the kidneys (22). This, thus far, remains a hypothesis, because no studies have ever quantitatively correlated the complications of PHPT or NHPT with dietary calcium intake. NHPT is still a relatively young disease in its definition (23), and the optimal amount of calcium intake is otherwise unknown in this variant.

For the aforementioned reasons, based on current clinical uncertainty on how much dietary calcium recommend to these patients, and due to lack of consistent and recent literature on this nutritional aspect of the disease, we aimed to characterize a population of patients with either PHPT or NHPT, compared to a control population, after excluding all other potential confounders, by examining dietary calcium intake evaluated by three independent validated questionnaires.

## 2 Patients and methods

This study was conducted in line with STROBE (STrengthening the Reporting of OBservational studies in Epidemiology) recommendations, and all patients consented to participate. The Ethical Committee of our Hospital (CE-AVEC) approved this study (protocol code: PARAT-AiS-BONE).

### 2.1 Study design and population

From February 1^st^, 2021, through April 31^st^, 2023, women with an established diagnosis of PHPT or NHPT and controls were consecutively enrolled at the Parathyroid and Bone Metabolism ambulatory of the Endocrinology and Prevention and Care of Diabetes Unit of IRCCS Azienda Ospedaliero-Universitaria of Bologna, Italy. Each patient meeting inclusion criteria (s. below) agreed to fill out and return 3 paper questionnaires on calcium intake within 1 week from the scheduled endocrine appointment.

The three questionnaires were all translated into Italian by the investigators, and were the following ones:

- The International Osteoporosis Foundation (IOF) food frequency questionnaire on calcium intake, otherwise called Calcium Calculator, print-made (24). The questions investigate the weekly consumption frequency of various calcium-containing foods (milk, yogurt, dairy products, meat, fish, eggs, starchy foods, fruit, vegetables).
- A local country-specific (LOC), previously validated, calcium intake questionnaire built on the Italian population (25). LOC was validated on women, and it considers typical Italian foods. Its main differences with the other questionnaires are the presence of a section dedicated to the consumption of calcium-rich bottled water, as well as the categorization of each food portion into small, medium, or large.
- The National Osteoporosis Foundation (NOF) questionnaire (26). This latter one was recommended in the US population by the NOF, and it estimates calcium intake through the daily amount of only three foods: milk, yogurt, and cheese. The total amount obtained is added with a standard quantity of 250 mg/day which can be either increased or reduced depending on the dietary habits reported by the patient, which represents the daily estimate of the amount of calcium taken with all the other foods in the diet.

### 2.2 Inclusion criteria

#### 2.2.1 Cases

Women with NHPT or PHPT. NHPT definition: NHPT defined as a persistently normal albumin-corrected and total serum calcium concentrations and persistently elevated PTH levels (at least 2 different determinations, at least 3 months apart between the determinations), after ruling out secondary causes of hyperparathyroidism such as renal disease (glomerular filtration rate [eGFR] < 60 mL/min), vitamin D deficiency (25-hydroxyvitaminD [25(OH)D] <20 ng/mL), and hypercalciuria (>400 mg/die).

PHPT definition: as per V International Workshop (2).

#### 2.2.2 Controls

Consecutive women referred from the Oncology Unit for their first bone density test and bone metabolism evaluation in the same recruiting period, because of recently diagnosed early-stage hormone-positive breast cancer. These women consented to fill out 3 paper questionnaires on calcium intake within 1 week from endocrine consultation.

Both cases and controls had a DXA performed within 6 months before the visit. All patients underwent the same laboratory tests in the LUM - Unified Metropolitan Laboratory of Bologna - within 1 month before the scheduled visit, as part of pre-specified medical protocols: PTH (reference range: 12 - 88 pg/mL), serum calcium (sCa) (reference range: 8.6 - 10.5 mg/dL), serum phosphate (reference range: 2.5 - 4.5 mg/dL), 25(OH)D (reference range: 20 - 100 ng/mL), albumin (reference range: 35 - 50 g/L), serum creatinine (reference range: 0.5 - 1.2 mg/dL), 24-hour urinary calcium (reference range: 50 - 400 mg/die), 24-hour urinary phosphorus (reference range: 0.4 - 1.3 g/die), serum bone alkaline phosphatase (reference range: 5.5 - 27.1 microg/L), serum C-terminal telopeptide of collagen type 1 (CTX) (reference range: 0.142 - 1.351 ng/mL). Abdominal ultrasound was carried out both in cases and controls, as part of endocrinological work-up (cases) or oncological assessment (controls).

### 2.3 Exclusion criteria

Any known condition that can affect bone and calcium metabolism, such as familial hypocalciuric hypercalcemia; malabsorption diseases, other diseases known to affect bone metabolism (thyrotoxicosis, bowel diseases, chronic hepatic disease, hypercortisolism, diabetes, rheumatological or hematological diseases, secondary hyperparathyroidism, previous parathyroidectomy); administration of drugs affecting bone and calcium metabolism (diuretics, lithium, bisphosphonates, denosumab, significant use of glucocorticoids within the past 2 years, chemotherapy or any treatment that could affect calcium metabolism), metabolic bone diseases such as Paget’s disease of the bone. Calcium supplements were among the exclusion criteria in either group.

### 2.4 Statistics

Absolute numbers and percentages were calculated for categorical data. The results for continuous variables were expressed as means and standard deviation (SD), minimum and maximum. A comparison of cases and controls was performed by Mann-Whitney U test. χ2 test was used to detect associations between categorical data. For each of the three questionnaires, multivariate analyses were used to identify predictors of serum calcium and PTH among patients with primary hyperparathyroidism, by adjusting for potential confounders. Non-normally distributed variables were log-transformed to be used in the multivariate analysis. The agreement between the three questionnaires was calculated with Cohen’s Kappa. Statistical analyses were performed using SPSS (version 26.0). P Values less than 0.05 were considered statistically significant.

## 3 Results

### 3.1 Clinical characteristics

The study population included 109 women: 54 patients with primary hyperparathyroidism (39 with PHPT and 15 with NHPT) and 55 controls (**Table 1**).

**Table 1:** Clinical characteristics of the whole population.

Variables	Controls N= 55	Primary Hyperparathyroidism Whole population N= 54	PHPT N= 39	NHPT N= 15
Age [years]	61.6 ± 10 [38; 82]	63.8 ± 10 [45; 81]	62.9 ± 10.8 [45; 81]	66.1 ± 7.2 [50; 77]
BMI [kg/m^2^]	25.8 ± 4 [18.4; 36.2]	26.5 ± 6.1 [16.7; 46.2]	26.1 ± 5.1 [18.6; 40.4]	27.3 ± 8.7 [16.7; 46.2]
Smoking history, n (%)	5 (9.1%)	4 (7.4%)	4 (10.3%)	0 (0.0%)
Daily alcohol assumption, n (%)	1 (1.8%)	0 (0%)	0 (0%)	0 (0%)
Menopause, n (%)	53 (96.4%)	48 (88.9%)	33 (84.6%)	15 (100%)
Age of menopause [years]	49.1 ± 4.1 [35; 56]	49.1 ± 4.9 [34; 56]	48.8 ± 5.2 [34; 56]	49.9 ± 4.2 [40; 55]
Disease duration [years]			1.8 ± 2.5 [0.0; 8.9]	4.5 ± 4.1 [0.0; 16.9] **^+^**
IOF Questionnaire [mg/day]	727 ± 219 [292; 1445]	688 ± 273 [231; 1412]	685 ± 290 [231; 1412]	695 ± 234 [303; 1287]
LOC Questionnaire [mg/day]	926 ± 348 [258; 1568]	859 ± 380 [274; 1940]	849 ± 386 [274; 1726]	887 ± 377 [362; 1940]
NOF Questionnaire [mg/day]	729 ± 207 [250; 1160]	709 ± 255 [320; 1450]	700 ± 256 [320; 1400]	733 ± 259 [350; 1450]
Cholecalciferol supplements, n (%)	40 (72.7%)	45 (83.3%)	30 (76.9%)	15 (100%)
Daily dosage of Cholecalciferol [IU]	1172 ± 378 [333; 2000]	1549 ± 635 [333; 3571]*	1387 ± 533 [333; 3300]	1875 ± 714 [1250; 3571] **^+^**
Clinical fractures, n (%)	4 (7.2%)	16 (29.6%)*	7 (17.9%)	5 (33.3%)
Nephrolithiasis, n (%)	2 (3.6%)	15 (27.8%)**	12 (30.8%)	3 (20.0%)
Normal bone density, n (%)	9 (16.4%)	7 (13.0%)*	7 (18%)	0 (0%)
Osteopenia, n (%)	29 (52.7%)	17 (31.5%)*	13 (33.3%)	4 (26.7%)
Osteoporosis, n (%)	17 (30.9%)	30 (55.5%)*	19 (48.7%)	11 (73.3%)

**P <.001 vs control group; *P <.05 vs control group; + P <.05 vs PHPT.

PHPT, Classical primary hyperparathyroidism; NHPT, Normocalcemic primary hyperparathyroidism; BMI, Body mass index; IOF, International Osteoporosis Foundation; LOC, Local; NOF, National Osteoporosis Foundation.

Mean age was comparable between cases and controls (63.8 years, 61.6 years, respectively), although NHPT patients were non-significantly older than PHPT patients (P=0.428). No differences were found between cases and controls in terms of body mass index (BMI), smoking habits, alcohol consumption and menopausal age.

Prevalence of renal stones (even microscopic) proved to be higher in patients with primary hyperparathyroidism (27.8%) compared to the control group (3.6%), as expected. Within the primary hyperparathyroidism group, prevalence of nephrolithiasis was non-significantly greater in PHPT than NHPT (30.8% vs. 20.0%, respectively).

Clinical fractures were more frequent in subjects with primary hyperparathyroidism compared to controls (29.6% vs 7.2%), but no differences were noted between the two variants of primary hyperparathyroidism. A previous fragility fracture was indeed reported in 17.9% of hypercalcemic patients and in 33.3% of NHPT patients.

As regards BMD, the most frequent diagnosis in the control group was osteopenia (52.7%), while osteoporosis was more common in primary hyperparathyroidism (55.5%). The prevalence of osteoporosis was non-significantly different between the two subgroups, with osteoporosis being diagnosed in 48.7% of PHPT patients and in 73.3% of NHPT patients.

The mean calcium intake estimated from the three validated questionnaires did not differ between primary hyperparathyroidism and controls, as well as between the two primary hyperparathyroidism variants. As regards vitamin D supplementation, it was comparable between the two subgroups, but NHPT took a higher daily dosage of cholecalciferol compared to PHPT patients (1875 IU vs 1387 IU). We also divided patients into three categories according to their calcium intake (**Supplementary Table 1**): very low (≤ 500 mg/die), low (500-1000 mg/die), sufficient (≥ 1000 mg/die) in order to calculate the agreement between the three questionnaires in identifying these three categories of calcium intake. A good level of concordance was found between IOF and NOF questionnaire (K=0.675, p<.001), while this was moderate between NOF and LOC questionnaire (K=0.451, p<.001), and low between IOF and LOC questionnaire (K=0.384, p<.001).

### 3.2 Biochemical and radiological characteristics

Regarding biochemical indices, as expected, mean PTH values, sCa, minimum and maximum serum calcium (sCaMin; sCaMax), albumin-corrected serum calcium, calcium/phosphate ratio (Ca/P ratio) and urinary calcium were higher in patients with primary hyperparathyroidism compared to controls, while serum phosphate was significantly lower. In the primary hyperparathyroidism group, bone alkaline phosphatase (BAP) and 25(OH)D levels were greater than in the controls. Urinary phosphate, CTX and renal function were similar between cases and controls (**Table 2**).

**Table 2:** Biochemical and radiological features of the whole population.

Variables		Controls N= 55	Primary Hyperparathyroidism Whole population N= 54	PHPT N= 39	NHPT N= 15
Serum Creatinine [mg/dl]		0.8 ± 0.1 [0.5; 1.1]	0.8 ± 0.2 [0.6; 1.4]	0.79 ± 0.18 [0.57; 1.44]	0.71 ± 0.12 [0.57; 0.99]
eGFR [mL/min]		83.3 ± 13.1 [60.0; 117.0]	83 ± 17 [39; 110]	82.4 ± 18.6 [39.0; 110.0]	85.7 ± 12.1 [66.0; 100.0]
Serum Calcium [mg/dL]		9.7 ± 0.4 [8.5; 10.7]	10.5 ± 0.7 [9.3; 12.8]**	10.8 ± 0.6 [9.7; 12.8]	10.0 ± 0.4 [9.3; 10.5]**^++^**
Serum Albumin [g/l]		42.0 ± 3.2 [31.7; 49.3]	43.2 ± 3.9 [36.8; 64.3]	43.4 ± 4.4 [36.8; 64.3]	42.5 ± 2.3 [37.0; 46.7]
Albumin-corrected Serum Calcium [mg/dL]		9.6 ± 0.3 [9.0; 10.4]	10.3 ± 0.7 [9.0; 12.6]**	10.5 ± 0.6 [9.4; 12.6]	9.8 ± 0.4 [9.0; 10.4]**^++^**
Serum Calcium Min [mg/dl]		9.4 ± 0.4 [8.5; 10.2]	10.0 ± 0.6 [8.9; 12.1]**	10.1 ± 0.6 [9.0; 12.1]	9.7 ± 0.5 [8.9; 10.4]**^+^**
Serum Calcium Max [mg/dl]		9.9 ± 0.4 [9.1; 10.8]	10.8 ± 0.6 [9.6; 12.8]**	11.1 ± 0.5 [10.3; 12.8]	10.1 ± 0.3 [9.6; 10.5]**^++^**
Serum Calcium Range [mg/dL]		0.47 ± 0.31 [0.0; 1.2]	0.80 ± 0.56 [0.0; 2.6]*	0.95 ± 0.56 [0.2; 2.6]	0.41 ± 0.32 [0.0; 1.3]**^++^**
Serum Phosphate [mg/dL]		3.8 ± 0.4 [2.6; 4.7]	3.1 ± 0.4 [2.1; 4.4]**	3.0 ± 0.5 [2.1; 4.4]	3.1 ± 0.4 [2.4; 3.7]
Serum Ca/P Ratio		2.6 ± 0.3 [2.0; 3.7]	3.5 ± 0.6 [2.4; 5.3]**	3.6 ± 0.6 [2.4; 5.3]	3.3 ± 0.5 [2.7; 4.3]**^+^**
Serum Magnesium [mg/dl]		2.0 ± 0.1 [1.8; 2.4]	2.1 ± 0.3 [1.5; 3.1]	2.1 ± 0.3 [1.5; 3.1]	2.0 ± 0.1 [1,8; 2.3]
Urinary Calcium [mg/24h]		162 ± 83 [38; 455]	253 ± 112 [35; 620]**	266 ± 119 [35; 620]	219 ± 84 [116; 384]
Urinary Phosphate [g/24h]		0.59 ± 0.25 [0.20; 1.50]	0.65 ± 0.27 [0.30; 1.40]	0.63 ± 0.26 [0.30; 1.40]	0.73 ± 0.29 [0.30; 1.30]
PTH [pg/mL]		48.3 ± 16.0 [18.0; 86.0]	108.8.8 ± 44.1 [37.0; 337.0]**	111.9 ± 50.7 [37.0; 337.0]	101.0 ± 16.4 [78.0; 147.0]
25(OH)D [ng/mL]		30.7 ± 11.3 [15.0; 66.0]	41.8 ± 26.7 [13.0; 188.0]**	43.2 ± 31.0 [13.0; 188.0]	38.1 ± 8.2 [21.0; 52.0]
CTX [ng/mL]		0.520 ± 0.294 [0.137; 1.574]	0.650 ± 0.393 [0.159; 1.980]	0.675 ± 0.436 [0.159; 1.980]	0.587 ± 0.257 [0.285; 1.123]
Bone Alkaline Phosphatase [microg/L]		19.7 ± 7.3 [7.9; 40.6]	24.0 ± 9.9 [9.7; 58.3]*	24.5 ± 11.3 [9.7; 58.3]	22.6 ± 5.5 [13.2; 32.9]
Lumbar Spine BMD		0.980 ± 0.139 [0.731; 1.246]	0.961 ± 0.165 [0.557; 1.273]	0.985 ± 0.168 [0.674; 1.273]	0.899 ± 0.142 [0.557; 1.112]
Lumbar Spine T-score		-1.684 ± 1.0 [-5.0; 0.6]	-1.8 ± 1.4 [-5.2; 0.8]	-1.6 ± 1.4 [-4.2; 0.8]	-2.3 ± 1.2 [-5.2; -0.6]
Lumbar Spine Z-score		-0.418 ± 1.0 [-2.1; 1.9]	-0.5 ± 1.2 [-2.9; 1.9]	-0.4 ± 1.2 [-2.9; 1.9]	-0.9 ± 1.1 [-2.7; 1.0]
TBS (L1-L4)		1.308 ± 0.126 [1.016; 1.627]	1.284 ± 0.132 [0.927; 1.505]	1.280 ± 0.143 [0.927; 1.505]	1.292 ± 0.107 [1.070; 1.476]
TBS (L1-L4) T-score		-1.8 ± 1.2 [-4.7; 0.9]	-2.0 ± 1.4 [-5.7; 0.2]	-2.1 ± 1.5 [-5.7; 0.2]	-1.8 ± 1.1 [-4.2; -0.1]
Femoral Neck BMD		0.784 ± 0.107 [0.540; 0.992]	0.762 ± 0.103 [0.554; 0.989]	0.775 ± 0.107 [0.554; 0.989]	0.730 ± 0.088 [0.564; 0.875]
Femoral Neck T-score		-1.6 ± 0.9 [-3.7; 0.1]	-1.8 ± 0.8 [-3.6; 0.1]	-1.7 ± 0.9 [-3.6; 0.1]	-2.1 ± 0.7 [-3.5; -0.9]
Femoral Neck Z-score		-0.4 ± 0.8 [-2.1; 1.1]	-0.5 ± 0.8 [-2.8; 1.2]	-0.5 ± 0.7 [-1.9; 1.2]	-0.6 ± 0.9 [-2.8; 0.8]
Total Hip BMD		0.847 ± 0.115 [0.630; 1.066]	0.817 ± 0.110 [0.587; 1.041]	0.826 ± 0.111 [0.598; 1.041]	0.795 ± 0.110 [0.587; 1.001]
Total Hip T-score		-1.3 ± 0.9 [-3.1; 0.6]	-1.5 ± 0.9 [-3.4; 0.3]	-1.4 ± 0.9 [-3.3; 0.3]	-1.7 ± 0.9 [-3.4; 0.0]
Total Hip Z-score		-0.3 ± 0.8 [-2.3; 1.4]	-0.4 ± 0.8 [-2.2; 1.3]	-0.4 ± 0.8 [-2.2; 1.3]	-0.5 ± 0.8 [-1.9; 1.0]
Ultradistal radius BMD		NA	0.383 ± 0.091 [0.196; 0.579]	0.399 ± 0.091 [0.196; 0.579]	0.343 ± 0.080 [0.226; 0.482]
Ultradistal radius T-score		NA	-1.8 ± 2.0 [-6.0; 2.6]	-1.4 ± 2.1 [-6.0; 2.6]	-2.6 ± 1.8 [-5.3; 0.4]
Ultradistal radius Z-score		NA	-0.5 ± 1.8 [-4.8; 4.3]	-0.3 ± 1.9 [-4.8; 4.3]	-1.0 ± 1.5 [-3.7; 1.8]
One-third distal radius BMD		NA	0.703 ± 0.132 [0.409; 0.998]	0.728 ± 0.139 [0.409; 0.998]	0.643 ± 0.094 [0.508; 0.869]**^+^**
One-third distal radius T-score		NA	-2.0 ± 1.5 [-5.3; 1.4]	-1.7 ± 1.6 [-5.3; 1.4]	-2.6 ± 1.1 [-4.2; -0.1]**^+^**
One-third distal radius Z-score		NA	-0.7 ± 1.2 [-4.2; 1.7]	-0.5 ± 1.3 [-4.2; 1.7]	-1.0 ± 1.0 [-2.7; 1.6]
Reason of the visit, n (%)	Hypercalcemia	NA	11 (20.4%)	11 (28.2%)	0 (0%)^+^
	Bone density screening	NA	18 (33.3%)	9 (23.1%)	9 (60.0%)**^+^**
	Vitamin D screening	NA	5 (9.3%)	3 (7.7%)	2 (13.3%)**^+^**
	Other reason	NA	20 (37.0%)	16 (41.0%)	4 (16.7%)**^+^**

**P <.001 vs control group; *P <.05 vs control group; ++P <.001 vs PHPT; + P <.05 vs PHPT.

PHPT, Classical primary hyperparathyroidism; NHPT, Normocalcemic primary hyperparathyroidism; eGFR, Estimated Glomerular Filtration Rate; Ca/P ratio, Calcium/Phosphate ratio; 25(OH)D, 25-hydroxyvitaminD; CTX, C-terminal telopeptide of collagen type 1; TBS, Trabecular bone score; NA, Not applicable.

Comparing the two variants of primary hyperparathyroidism, mean sCa levels were higher in PHPT (10.8 mg/dL) compared to NHPT (9.9 mg/dL), as were sCaMin and sCaMax, serum calcium corrected for albumin, and Ca/P ratio, as expected. The numerical difference between sCaMax and sCaMin, called serum calcium range (sCaRange), was higher in PHPT than NHPT (0.95 mg/dL vs 0.45 mg/dL). The remaining biochemical variables were similar between the two variants.

No significant differences in BMD T-scores were found between patients with primary hyperparathyroidism and the control group. Comparing the variants, it emerged that NHPT had a significantly worse BMD and T-score at one-third distal radius compared to the hypercalcemic variant, while lumbar spine and femoral neck were comparable.

Another important difference was found in the endocrine consultation reason. In fact, the most common reason that led NHPT patients to seek a specialist evaluation, which subsequently resulted in the diagnosis of primary hyperparathyroidism, was bone metabolism screening (60.0%). By contrast, as regards PHPT, the diagnosis was most frequently made incidentally (41.0%) during other endocrinological investigations (e.g., evaluation of thyroid nodules, or other reasons).

### 3.3 Multivariate analyses

A multivariate regression analysis was performed to determine predictive factors of SCa in patients with primary hyperparathyroidism (**Table 3**). Independent of confounding factors (25(OH)D, age and renal function), sCa was not significantly associated with calcium intake estimated by the three questionnaires,.

**Table 3:** Multivariate analysis of serum calcium in the primary hyperparathyroidism cohort.

Dependent variable	Serum calcium [mg/dL]					
	IOF Questionnaire		LOC Questionnaire		NOF Questionnaire	
	β [95%CI]	P value	β [95%CI]	P value	β [95%CI]	P value
Intercept	11.780 [9.652; 13.908]	**< 0.001**	11.698 [8.298; 15.097]	**< 0.001**	11.904 [7.951; 15.858]	**< 0.001**
Predictive factors						
Age [years]	-0.017 [-0.039; 0.004]	0.114	-0.016 [-0.038; 0.005]	0.137	-0.016 [-0.038; 0.006]	0.146
25(OH)D [ng/mL]	0.005 [-0.003; 0.012]	0.227	0.004 [-0.003; 0.011]	0.279	0.004 [-0.004; 0.012]	0.304
Calcium intake [mg/day]	0.000 [0.000; 0.001]	0.483	0.032 [-0.395; 0.458]	0.881	-0.001 [-0.533; 0.530]	0.997
eGFR [mL/min]	-0.006 [-0.019; 0.007]	0.362	-0.006 [-0.019; 0.007]	0.365	-0.006 [-0.019; 0.007]	0.356

25(OH)D, 25-hydroxyvitaminD; eGFR, Estimated Glomerular Filtration Rate.

Specifications: Calcium intake estimated from LOC and NOF were considered as natural logarithm of the variable. Significant P-values are highlighted in bold.

A similar analysis was carried out to evaluate predictors of PTH levels in patients with primary hyperparathyroidism (**Table 4**), adjusted for cholecalciferol supplements, which revealed that renal function and dietary calcium intake estimated from the questionnaires significantly affected PTH. Older age both in PHPT and NHPT variant was also a significant negative predictor, by contrast 25(OH)D was not a significant predictor of PTH levels in this study population.

**Table 4:** Multivariate analysis of serum PTH in the primary hyperparathyroidism cohort.

Dependent variable	Serum PTH [pg/mL]					
	IOF Questionnaire		LOC Questionnaire		NOF Questionnaire	
	β [95%CI]	P value	β [95%CI]	P value	β [95%CI]	P value
Intercept	394.478 [276.926; 512.031]	**< 0.001**	528.623 [334.803; 722.443]	**< 0.001**	623.534 [412.919; 834.150]	**< 0.001**
Predictive factors						
Age [years]*PHPT	-1.705 [-2.919; -0.490]	**0.006**	-1.841 [-3.040; -0.642]	**0.003**	-1.659 [-2.829; -0.489]	**0.005**
Age [years]*NHPT	-1.736 [-2.907; -0.565]	**0.004**	-1.850 [-3.008; -0.692]	**0.002**	-1.670 [-2.801; -0.539]	**0.004**
25(OH)D [ng/mL]	-0.179 [-0.647; 0.289]	0.454	-0.131 [-0.591; 0.330]	0.578	-0.255 [-0.715; 0.205]	0.277
Calcium intake [mg/day]	-0.039 [-0.077; -0.002]	**0.040**	-23.977 [-47.432; -0.522]	**0.045**	-38.833 [-66.159; -11.507]	**0.005**
eGFR [mL/min]	-1.689 [-2.419; -0.960]	**< 0.001**	-1.630 [-2.356; -0.904]	**< 0.001**	-1.735 [-2.441; -1.028]	**< 0.001**

PHPT, Classical primary hyperparathyroidism; NHPT, Normocalcemic primary hyperparathyroidism; 25(OH)D, 25-hydroxyvitaminD; eGFR, Estimated Glomerular Filtration Rate.

Specifications: Calcium intake estimated from LOC and NOF were considered as natural logarithm of the variable; the results were adjusted by cholecalciferol supplements.Significant P-values are highlighted in bold.

## 4 Discussion

The present study represents an important survey of dietary calcium intake in primary hyperparathyroidism. Furthermore, it is the first study on primary hyperparathyroidism to include NHPT with a novel standardized evaluation of calcium intake. The methodology used to quantify dietary calcium intake through three validated questionnaires was robust, unlike previous studies in which food diaries were used. The administration of FFQs is simpler for patients and clinicians and could potentially be replicated in other studies. Also, FFQs have a high specificity in identifying subjects with a low calcium intake.

### 4.1 Dietary calcium intake in PHPT

Optimal dietary calcium intake is fundamental in the homeostasis of bone and renal metabolism in healthy individuals and even more so in primary hyperparathyroidism. Dietary management of patients with primary hyperparathyroidism treated conservatively remains a subject of debate, as the data available to date are limited. Previous studies (12–16) that analyzed dietary calcium intake and its associations with densitometric, and laboratory indices of bone metabolism were conducted on a limited number of patients with PHPT and essentially date back to 1980s and 1990s. In addition, these studies were conducted with different methodologies and their findings tended to be discordant. For instance, the longitudinal study conducted in 1997 by Gartenberg et al. (13) evaluated the effect of calcium intake in 71 unselected patients with asymptomatic PHPT, to investigate whether the latter was associated with changes in biochemical parameters and BMD. The subjects reported their diet in a food diary for one week, and according to the average daily intake of dietary calcium the patients were divided into three groups: very low intake (<300 mg/day), low (300-500 mg/day) and US RDA (> 800 mg/day). The results showed no significant effects of calcium intake on serum calcium, PTH, serum phosphate, 25(OH)D, 1,25(OH)_2_D, urinary calcium excretion, and BMD at the different sites, although in the group with low dietary calcium intake (300-500 mg/day), patients with elevated serum 1,25(OH)_2_D had higher levels of PTH and urinary calcium. The Authors concluded that patients with normal 1,25(OH)_2_D levels could liberalize their calcium intake without adverse consequences, but patients with elevated 1,25(OH)_2_D levels should restrict their calcium intake in order to prevent hypercalciuria. By contrast, Insogna et al. in 1985 (12), in a study of 18 unselected PHPT patients, reported that a diet containing a normal-to-high calcium intake (>1000 mg/day) was associated with a slight increase in urinary calcium excretion (from 281 to 337 mg/day) and a significant reduction in the level of circulating PTH.

The impact of dietary calcium intake on the pathophysiology of PHPT is not yet clear. In fact, according to the latest data in literature, a recent commentary by Rao et al. (27) underlines the importance of Vitamin D and calcium nutrition in sporadic parathyroid tumorigenesis, hypothesizing that restriction of calcium intake would promote adenoma growth in patients with sporadic PHPT, and according to the aforementioned Nurses’ Health Study I (18), it would appear to be a risk factor for sporadic PHPT in healthy women.

Moreover, optimal dietary calcium intake in patients with primary hyperparathyroidism represents a diagnostic cornerstone, especially in NHPT to exclude secondary hyperparathyroidism (8, 9). A recent narrative review (28) reinforces the importance of following U.S. Institute of Medicine guidelines (17) because dietary calcium intake and intestinal absorption commonly lead to variability in laboratory measurements due to day-to-day variation, making it more difficult to confirm a precise diagnosis during daily clinical practice.

According to the systematic review of Balk et al. (29) on global dietary calcium in adults, in Italy the average intake was estimated to be 765 mg/day, that is in line with what was reported in the present study by each of the three validated questionnaires both in cases and controls. Considering data by Palermo et al. (30), in which the IOF questionnaire was used to estimate calcium intake in NHPT (798 ± 89 mg) and PHPT (777 ± 95 mg), the results of our study were similar (NHPT: 686 ± 216 mg; PHPT: 685 ± 290 mg). Because there were no differences in the three questionnaire estimates, during daily clinical practice we would recommend using NOF questionnaire because, by investigating only three fundamental items (milk, yogurt and cheese), it might be less time-consuming and more intuitive for patients, while still guaranteeing a reliable estimate of dietary calcium intake.

### 4.2 Clinical characteristics

By analyzing the clinical features of primary hyperparathyroidism, the present study confirmed that the prevalence of kidney stones was significantly higher in primary hyperparathyroidism compared to controls. This agrees with previous studies on renal manifestations (31, 32), which reported nephrolithiasis as one of the most common complications of primary hyperparathyroidism with an estimated prevalence of 15-20%, in the present study being 27.8%.

The difference between clinical fractures proved to be significant, with a higher prevalence in primary hyperparathyroidism compared to controls (29.6% vs 7.2%) and in this regard, epidemiological studies have shown that primary hyperparathyroidism was associated with an increased risk of both vertebral and peripheral fractures (33, 34).

In NHPT patients, as expected, cholecalciferol supplementation was greater than in patients with the classic variant of PHPT because, by definition, other secondary causes of parathyroid hormone elevation must be excluded when serum calcium is within normal limits (5). Among these causes is hypovitaminosis D, thereby justifying the greater need for supplementation in NHPT patients compared with hypercalcemic PHPT, in which it is sufficient to highlight the inappropriate secretion of PTH in the setting of hypercalcemia regardless of 25(OH)D levels (2).

### 4.3 Biochemical and radiological characteristics

Biochemistries were similar between the two variants, except for sCa, which was the only key parameter for differentiating the two variants. As expected, serum Ca/P ratio was higher in the whole population with primary hyperparathyroidism compared with controls, confirming what was previously noted by Madeo et al. (35). Serum levels of PTH, 25(OH)D and renal function were comparable between the two subtypes. These findings also agree with the cross-sectional multicenter study by Palermo et al. (30), where 47 NHPT and 41 PHPT patients were compared, and no significant differences were found in the aforementioned variables. Our data on renal function also confirmed what was highlighted in the prospective study by Diaz-Soto et al. (36), although data on PTH and 25(OH)D were discordant. Indeed, in that study (36) 61 patients with NHPT and PHPT were compared, finding that in the NHPT group PTH values were lower, while 25(OH)D levels were higher. Regarding urinary calcium excretion, the present study and those by Diaz-Soto et al. (36) and Kiriakopoulos et al. (37), found no significant differences between NHPT and PHPT, as opposed to the study by Palermo et al. (30), in which NHPT showed lower urinary calcium excretion than PHPT.

As expected, densitometric diagnosis differed significantly between case and control patients. Interestingly, a non-significantly higher percentage of osteoporosis was detected in NHPT (73,3%) compared to PHPT (48,7%), as reported by other studies (38–40). Also, regarding densitometric indices, NHPT patients showed their BMD and T-scores of the one-third distal radius to be lower than those of PHPT patients, but BMD and T-scores of femoral neck, total hip, lumbar spine and TBS were similar. These findings partially agree with a recent retrospective chart review by Wei Shan Hoong et al. (10) where 73 NHPT and 84 PHPT were compared, confirming no differences in TBS and femoral neck BMD. By contrast, it reported a lower BMD and T-scores of total hip and lumbar spine in NHPT. To sum up the normocalcemic variant apparently showed worse densitometric profile at one-third distal radius. This finding confirms the ascertainment bias hypothesis in NHPT, because in these patients PTH was ordered for low BMD not for suspicion of PHPT. NHPT is often diagnosed during the evaluation of patients with low bone mass or osteoporosis (38, 41–43). This also occurred in the present study, the most frequent reason for NHPT diagnosis being osteopenia or osteoporosis screening. Another source of selection bias might be that NHPT is often diagnosed after a clinical event (such as a fragility fracture or nephrolithiasis) in third-level reference centers for metabolic bone diseases (5, 7).

### 4.4 Impact of dietary calcium intake

Considering multivariate analyses, irrespective of the other confounding factors, we found that sCa was not influenced by dietary calcium intake nor by levels of 25(OH)D, thereby suggesting that it is the intrinsic variant of primary hyperparathyroidism which determines the setpoint of sCa.

By contrast, dietary calcium intake significantly affected PTH values, in particular a higher intake was associated with lower PTH levels and vice versa. Our data on PTH predictors confirmed what was reported in the study by Insogna et al. (12), according to which a diet containing a sufficient intake of calcium in unselected PHPT was associated with a significant PTH reduction, but contrasted with what was reported in the study by Gartenberg et al. (13), which did not highlight a significant effect of dietary calcium on PTH. However, it is worth specifying that the normocalcemic variant was not taken into consideration in these studies, as it was officially recognized as a subtype of PHPT after completion of these studies (23). In this regard, NHPT patients had a comparable calcium intake to those with PHPT. According to multivariate analyses, calcium intake does not seem to influence this variant, the same applies to 25(OH) vitamin D levels, once these are maintained sufficient. In fact, even if NHPT patients took a higher cholecalciferol supplementation than PHPT, serum calcium was persistently normal.

As a result, it seems reasonable that patients with primary hyperparathyroidism with low calcium intake may benefit from increasing calcium through diet or supplements to reach the recommended daily allowances. This hypothesis was also tested by Jorde et al. (14) in a 1-year open study in 31 patients with asymptomatic PHPT. In this case, dietary calcium intake was calculated through the administration of a questionnaire that investigated the consumption of 42 different foods and patients with a calcium intake < 450 mg/day received a daily supplement of 500 mg of elemental calcium, while those with an intake > 450 mg/day were followed without any intervention. In the first group, a transient and non-significant increase in sCa at 4 and 12 weeks was detected, as well as a transient but significant increase in BMD at femoral neck at the end of the study. Serum phosphate and 24-hour calcium excretion were not affected.

Considering the data shown by our study it seems evident that restriction of calcium intake has no place in the conservative management of primary hyperparathyroidism, instead, our study favors adequate dietary calcium intake to mitigate PTH levels, regardless of the variant of PHPT. Moreover, calcium intake optimization might be fundamental in potentially slowing down PTH-related complications, both from the perspective of conservative and surgical therapy. Assessing and recommending the right amount of calcium intake should represent one of the first therapeutic approaches in primary hyperparathyroidism, because calcium deficiency is easily detectable through validated questionnaires.

The single-center case-control design and the availability of centralized clinical, laboratory, nutritional, and densitometric variables are among the strengths of our study. Moreover, the standardized methodology to evaluate calcium intake makes the study robust and replicable in other larger settings. The study also had some limitations: the cross-sectional design prevents us from proving causality in the associations found in the primary hyperparathyroidism group. Also, ionized calcium was not assessed, although albumin-corrected serum calcium was tested on every scheduled laboratory evaluation both in cases and controls. The limited sample of NHPT patients also prevented us from carrying out more powerful analyses.

## 5 Conclusions

Primary hyperparathyroidism patients should not restrict their dietary calcium intake, because calcium intake was found to be negatively associated with PTH levels, independent of other clinical parameters.

Serum calcium does not appear to be significantly influenced by dietary calcium intake and 25(OH)D concentration in primary hyperparathyroidism. Considering these results the patients, particularly those with low dietary calcium intake, can therefore be safely encouraged to meet calcium intake requirements in order to reach allowances recommended in the general population, without significant risk of hypercalcemia.

Except for serum calcium, NHPT as compared with PHPT seemed to have a similar biochemical profile, while it would appear to have a worse densitometric profile at one-third distal radius. The occurrence of nephrolithiasis and clinical fragility fractures were otherwise similar between variants.

Further longitudinal studies could be useful to confirm these data and to evaluate whether dietary calcium intake evaluated with a standard methodology such as FFQs could positively influence bone density and biochemistries over time, especially in patients with primary hyperparathyroidism not referred to surgery or in whom surgery is contraindicated.

## Data Availability

The raw data supporting the conclusions of this article will be made available by the authors, without undue reservation.
