# Supplementary material for: Dietary calcium intake in primary hyperparathyroidism and in its normocalcemic variant: a case-control study
Source: Front Endocrinol (Lausanne). 2025 Feb 3;15:1428640. doi: 10.3389/fendo.2024.1428640 (PMC11830599; doi:10.3389/fendo.2024.1428640)
Supplement: Supplementary file 1 [file DataSheet1.docx]

Supplementary Material

# Supplementary Figures and Tables

**Table 1 Supplementary.** Agreement between questionnaires on calcium intake.

| **IOF and LOC questionnarire** | | **IOF and NOF questionnarire** | | **NOF and LOC questionnarire** | |
| --- | --- | --- | --- | --- | --- |
| **Cohen’s K** | **P Value** | **Cohen’s K** | **P Value** | **Cohen’s K** | **P Value** |
| 0.384 | <.001 | 0.675 | <.001 | 0.451 | <.001 |
| Abbreviations: IOF, International Osteoporosis Foundation; LOC, Local; NOF, National Osteoporosis Foundation.  Specifications: Patients were divided into three categories according to calcium intake: very low (≤ 500 mg/die), low (500-1000 mg/die), sufficient (≥ 1000 mg/die). | | | | | |

**Table 2 Supplementary.** International Osteoporosis Foundation (IOF) Questionnaire

| **First name** |  |  |
| --- | --- | --- |
| **Surname** |  |  |
| **Date of birth** |  |  |
| **Questionnaire delivery date** |  |  |
| **FOOD** | **QUANTITY** |  |
|  |  | **BRAND OF SUPPLEMENT AND NUMBER OF DOSES (sachets, tablets.. )** |
| **Calcium supplements** | 500mg |  |
|  |  | **NUMBER OF PORTIONS PER WEEK** |
| **Types of Milk** |  |  |
| Milk | 200ml |  |
| Milkshake | 300ml |  |
| Sheep's milk | 200ml |  |
| Coconut milk | 200ml |  |
| Soya drink ( enriched ) | 200ml |  |
| Soya milk | 200ml |  |
| Rice milk | 200ml |  |
| Avena's milk | 200ml |  |
| Almond milk | 200ml |  |
|  |  |  |
| **Yogurt** |  |  |
| Flavored yogurt | 150mg |  |
| Yogurt with fruit pieces | 150mg |  |
| Plain yogurt | 150mg |  |
|  |  |  |
| **Cheese** |  |  |
| Hard cheese (e.g. Cheddar, Gruyere, Emmental, Parmesan) | 30g |  |
| Soft cheese (e.g. Camembert, Brie) | 60g |  |
| Feta | 60g |  |
| Mozzarella cheese | 60g |  |
| Fresh cheese (e.g. cottage cheese, ricotta, mascarpone) | 200g |  |
| Cheese cream | 30g |  |
|  |  |  |
| **Cream, sweets** |  |  |
| Whipped Cream | 30ml |  |
| Cooking cream | 30ml |  |
| Vanilla custard | 120g |  |
| Vanilla ice cream | 100g |  |
| Vanilla pudding | 120g |  |
| Rice pudding | 200g |  |
| Pancake | 80g |  |
| cheesecake | 200g |  |
| Waffle | 80g |  |
|  |  |  |
| **Meat, Fish, Eggs** |  |  |
| Egg | 50g |  |
| Red meat | 120mg |  |
| Chicken | 120mg |  |
| Fish (e.g. cod, trout, herring, whitebait) | 120mg |  |
| Canned tuna | 120mg |  |
| Sardines in oil (canned) | 60mg |  |
| Smoked salmon | 60mg |  |
| Shrimps | 150mg |  |
|  |  |  |
| **Legumes** |  |  |
| Lentils | 80g raw, 200g cooked |  |
| Chickpeas | 80g raw, 200g cooked |  |
| Beans | 80g raw, 200g cooked |  |
| Red beans | 80g raw, 200g cooked |  |
|  |  |  |
| **Farinaceous** |  |  |
| Pasta (cooked) | 180g |  |
| Boiled rice) | 180g |  |
| Boiled potatoes) | 240g |  |
| White bread (1 slice) | 40g |  |
| Wholemeal bread (1 slice) | 40g |  |
| Muesli (cereals) | 50g |  |
| Naan | 60g |  |
|  |  |  |
| **Fruit** |  |  |
| Orange | 150g |  |
| Apple | 120g |  |
| Banana | 150g |  |
| Apricots (3 fruits) | 120g |  |
| Dried currants or gooseberries | 120g |  |
| Dried figs | 60g |  |
| Raisins | 40g |  |
|  |  |  |
| **Vegetables** |  |  |
| Lettuce | 50g |  |
| Cabbage | 50g raw |  |
| Chinese cabbage | 50g raw |  |
| Broccoli | 120g raw |  |
| Okra/ Okra | 120g raw |  |
| Watercress | 120g |  |
| Rhubarb | 120g raw |  |
| Carrots | 120g raw |  |
| Tomatoes | 120g raw |  |
|  |  |  |
| **Dried fruit and seeds** |  |  |
| Almonds | 30g |  |
| Nuts | 30g |  |
| Hazelnuts | 30g |  |
| Brazil nuts | 30g |  |
| Sesame | 15g |  |
| Tahini | 30g |  |
|  |  |  |
| **Cooked dishes** |  |  |
| Quiche (with eggs and cheese) | 200g |  |
| Cheese omelette | 120g |  |
| Pasta with “Alfredo” cheese | 330g |  |
| Pizza | 300g |  |
| lasagna | 300g |  |
| Cheeseburger | 200g |  |
|  |  |  |
| **Other foods** |  |  |
| Tofu | 120g |  |
| Algae | 100g |  |
| Wakame | 100g |  |

**Figure 1 Supplementary.** Italian calcium intake (LOC) questionnaire


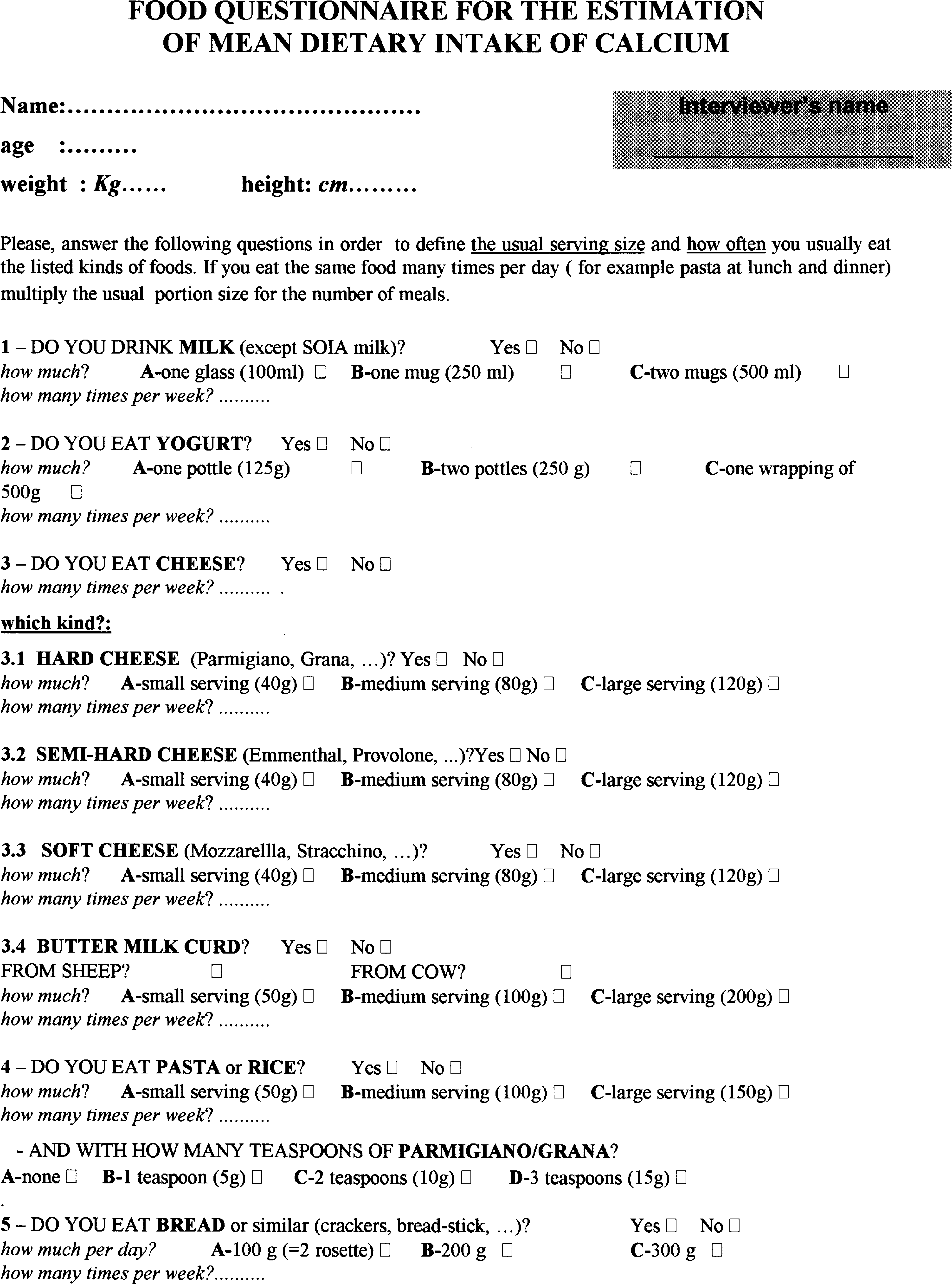


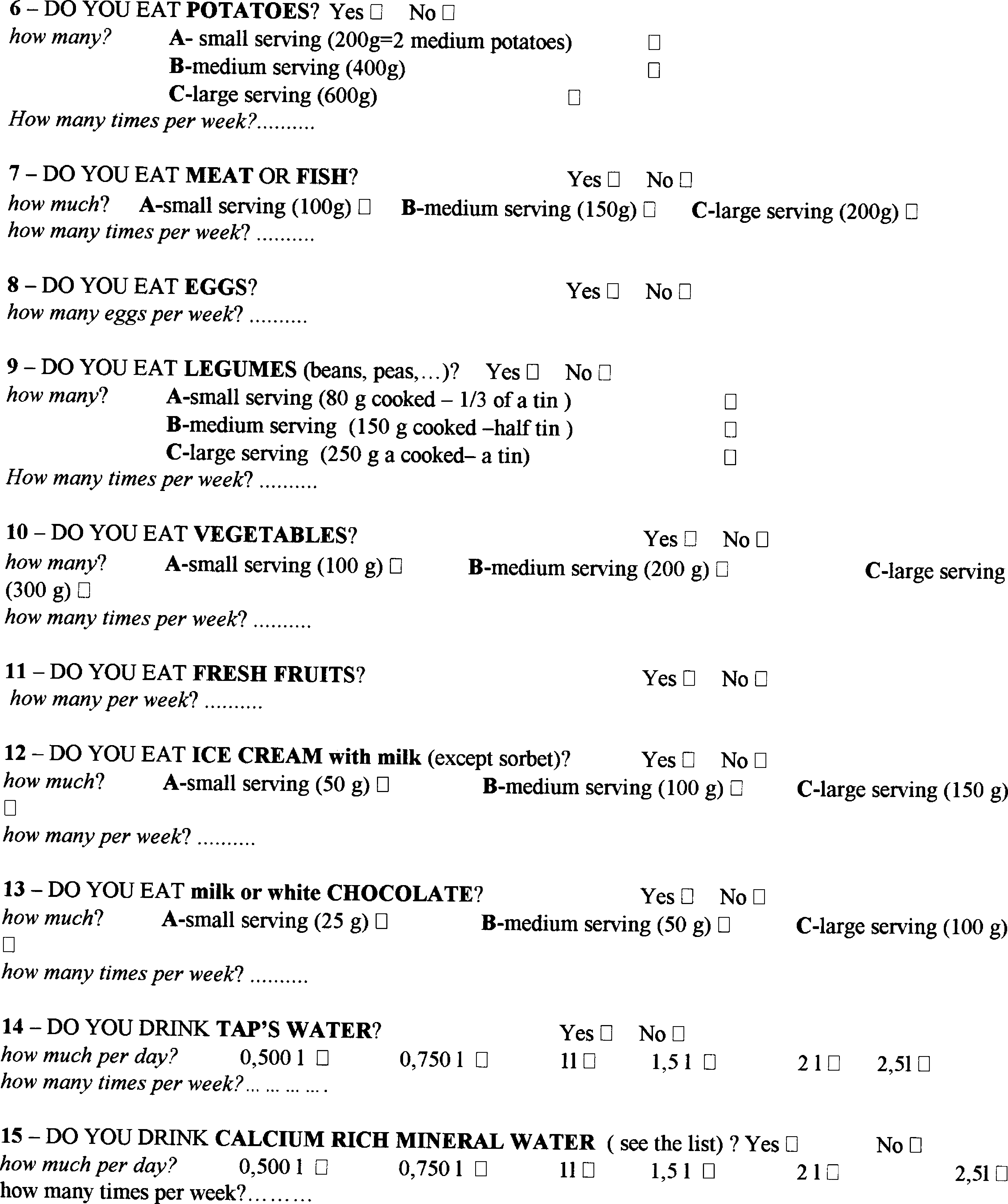


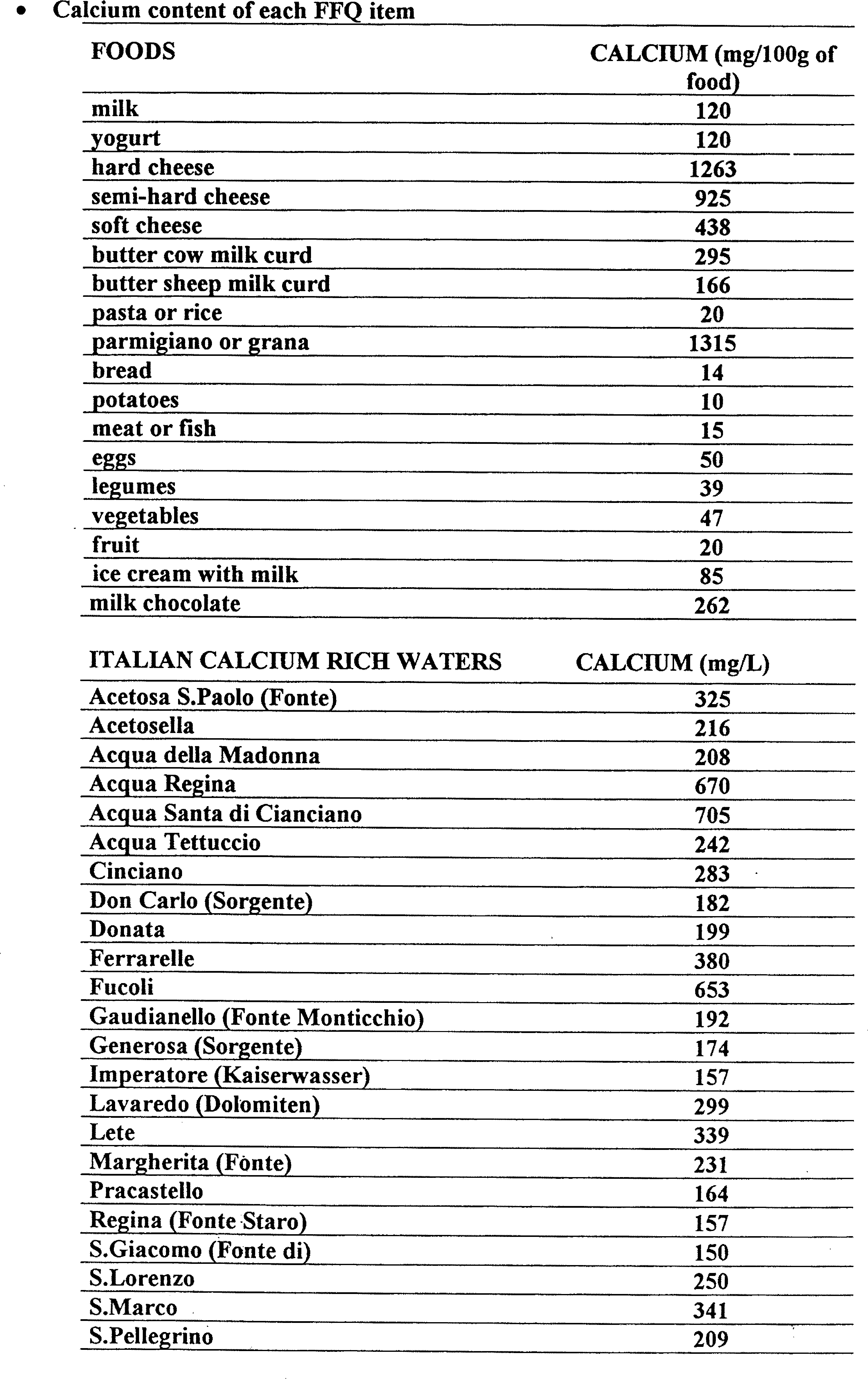


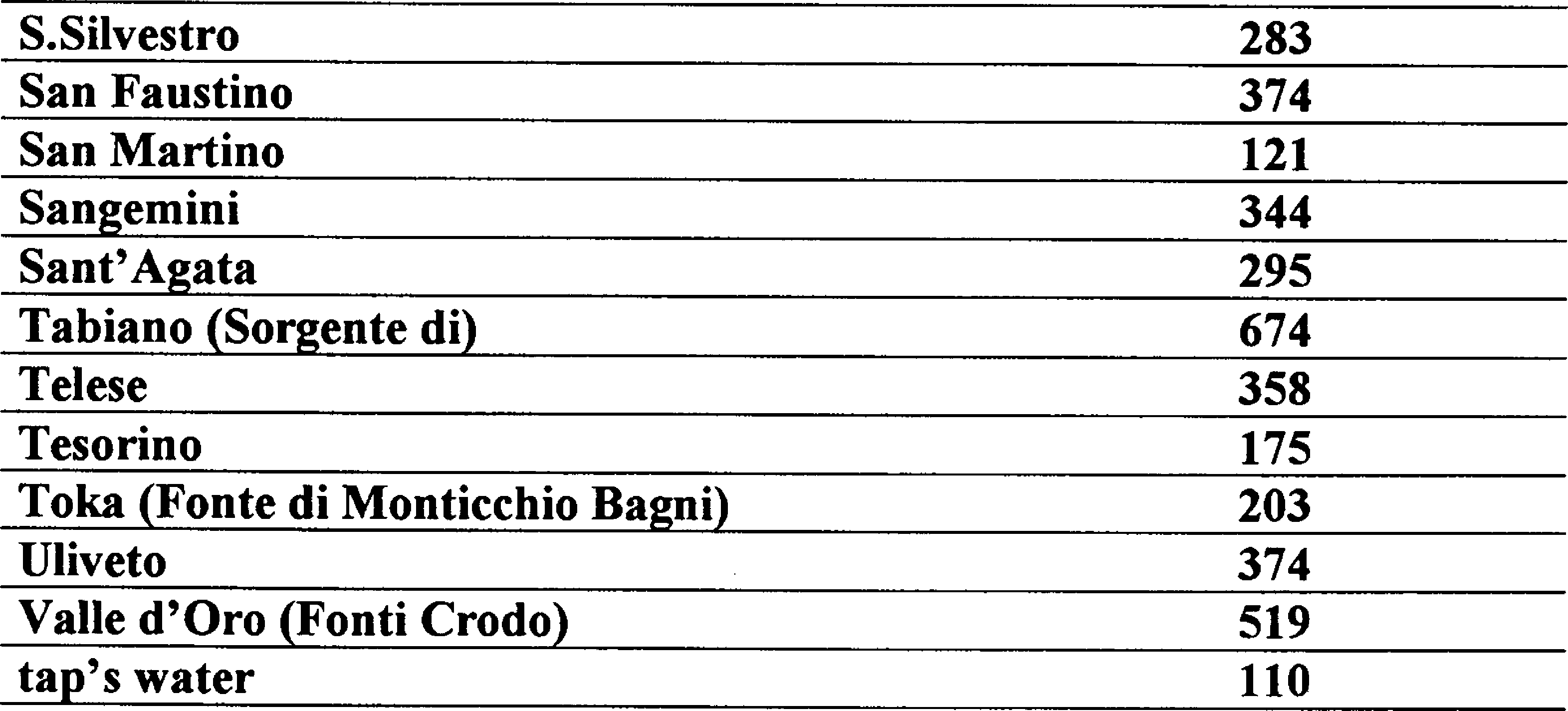


**Table 3 Supplementary.** National Osteoporosis Foundation (NOF) questionnaire

| Food | How many portions do you take per day? |
| --- | --- |
| Milk (220 ml) | .................................................. ........................................ |
| Yogurt (170 g) | …………………............................................................................ |
| Cheese (30 g) | .................................................. ....................................... |
| Estimate from other foods (increase this value if the patient eats more than 250g from other foods) | 250 g |
